# Full-Section Deformation Monitoring of High-Altitude Fault Tunnels Based on Three-Dimensional Laser Scanning Technology

**DOI:** 10.3390/s24082499

**Published:** 2024-04-13

**Authors:** Dongmei Tan, Yu Tao, Baifeng Ji, Qinlin Gan, Tai Guo

**Affiliations:** 1 School of Civil Engineering and Architecture, Wuhan University of Technology, Wuhan 430070, China; taoyu123456@whut.edu.cn (Y.T.); jbfeng@whut.edu.cn (B.J.); 15335881979@163.com (Q.G.); gt32882323@163.com (T.G.); 2 Sanya Science and Education Lnnovation Park, Wuhan University of Technology, Sanya 572000, China

**Keywords:** three-dimensional laser scanner, tunnel, deformation monitoring, complex surface structure

## Abstract

In traditional tunnel monitoring, the characteristic points of an object within a tunnel are measured to obtain information about the object. Considering the limitations of the traditional method in measuring the complex surface structure of tunnels, such as limited monitoring points, a long measurement period, and low precision, this study introduces an approach that uses three-dimensional (3D) laser scanning for monitoring tunnel cross-section deformation. Using this approach, the soft surrounding rock of a high-altitude ultralong tunnel was taken as the monitoring object. The test tunnel was first scanned using a 3D laser scanner, and the collected data were processed. The internal structural data of the tunnel were subsequently compared with its actual contour lines and the data of its primary branch and secondary lining on different dates. The results indicate that the arch roof of the tunnel tended to be stable within a certain time range when the positions of the primary branch and secondary lining were at different measuring points with different pile numbers. The deformation of the pile number on the left and right sides did not generally exceed 0.02 m, except at a few measuring points. A comparison between the actual cross section of the initial branch and that of the designed section showed that the actual elevation of the arch of the initial branch of the tunnel was greater than its designed elevation by no more than 0.3 m. Hence, through this study, a convenient and practical method is presented for monitoring deformation in complex curved tunnel structures.

## 1. Introduction

Urban rail transit is an important means of passenger transportation. Tunnel construction has developed rapidly in metropolises because tunnels can significantly improve the traffic efficiency and convenience of urban rail transit systems. The safety monitoring of tunnel structures during tunneling operations is required to ensure the safety of life and property [1]. However, tunnel deformation has become a main factor affecting tunnel safety. Tunnel deformation is typically caused by changes in environmental stress. Thus, to ensure the safety of a tunnel, its deformation has to be closely monitored. When the deformation exceeds a certain threshold, typically a few centimeters, the overall safety of the tunnel will be seriously threatened [2]. The detection of structural deformation and hazards in tunnel construction involves a significant workload [3]. The traditional methods currently being used for tunnel deformation monitoring and measurement still use total stations and convergence instruments. However, construction site environments are now complex, and the distribution of the target points to be measured follow both regular and irregular surface patterns; thus, complete data acquisition on the surface of a tunnel structure using traditional deformation monitoring methods is challenging [4,5]. Due to the limitation of the number of monitoring points, these traditional methods can only measure the deformation of a very limited set of points, resulting in insufficient monitoring precision, and will not provide a complete tunnel wall model [6], which has certain limitations. Three-dimensional (3D) laser scanning is one of the most rapidly developed automatic displacement monitoring methods used in the past two decades [7], compensating for the shortcomings of traditional measurement methods [8,9,10]; it has the advantages of high precision, high efficiency, and high automation, and can obtain the deformation information of tunnel structures comprehensively, quickly, and precisely. The 3D scanners currently in use are based on the principle of laser ranging. By calculating the 3D coordinates of a large number of dense points on the surface of an object to be measured by recording its distance from the measuring device, horizontal and vertical angles, and reflectivity, the 3D model of the object can be quickly reconstructed along with the associated diagram data, such as lines, surfaces, and bodies [11,12,13,14]. Because 3D laser scanning can obtain the surface information of objects with a panoramic view, high precision, and fast speed, it is widely used in various civil engineering applications such as automated modeling, construction progress tracking, construction safety management, and automated construction [15,16,17,18,19,20,21,22,23,24]. Moreover, 3D laser scanners can be used in disaster prevention and reduction applications to monitor and calculate landslide, rock fall, bank collapse, and mine collapse deformations in dangerous and difficult-to-reach locations.

In recent years, 3D laser scanning has been widely used for tunnel monitoring and measurements. Many scholars have made significant contributions to the research conducted on 3D laser scanning. Wu et al. [25] conducted long- and short-term monitoring of tunnels using 3D laser scanning technology. The temporal and spatial thinning characteristics of deformation at the representative points of the middle section, the working face of the roadway, and the mileage of the tunnel were analyzed during three-step and seven-step excavation. Zhang et al. [26] proposed a tunnel structure monitoring scheme based on a new 3D laser measurement technology designed for mobile applications. With 3D laser scanning technology gaining popularity in an increasing number of fields, tunnel deformation monitoring [27,28,29,30,31], point-cloud denoising [32], and tunnel section extraction and analysis [33,34] have become the main focus of studies on this topic. Deformation-fitting analysis and various visualization algorithms used in 3D laser scanning employed for tunnel detection are also receiving increasing attention. Van et al. [35] projected a tunnel point cloud onto a cylinder and obtained a 3D regular mesh of the tunnel to measure its deformation. Zhu [36] proposed a method for the deformation monitoring of tunnels using 3D laser scanning point clouds to intercept tunnel cross sections and fit them with ellipses. Lindenbergh et al. [37] proposed a deformation monitoring method based on a laser point-cloud data-fitting circle and tested the feasibility of using a newly introduced panoramic high-precision laser scanner (Leica HDS3000) for tunnel deformation monitoring; compared to the single-point measurement method, the proposed method had higher analysis precision. However, the deformation section was no longer elliptical. Walton et al. [38] and Li et al. [39] successfully performed good deformation analyses by fitting the ellipse as a whole. Xie et al. [40] applied laser-scanning technology to tunnel deformation monitoring and studied a point-cloud processing method. They proposed a 3D modeling algorithm for a tunnel point cloud to address the relative deformation of the tunnel point cloud. The reliability of the algorithm was verified through field experiments. Hu et al. [41] processed 3D laser scanning point-cloud data using the Kriging filtering algorithm. The point-cloud data were extracted and analyzed through field monitoring of the test section, and the deformation data obtained from the test were compared with the measurement data obtained using the traditional method to verify the precision of the algorithm.

Unlike traditional methods, 3D laser scanning can easily obtain high-density and high-precision observation data with a high sampling rate, effectively enabling complex curved tunnel structure measurements. Thus, 3D laser scanning is given priority in tunnel deformation monitoring. The main focus of ongoing research is on the deformation monitoring and the deformation algorithm of subway tunnels and the deformation characteristics of tunnel cross sections. However, the applicability of 3D laser scanning technology for monitoring highway tunnel deformations remains unclear. Therefore, deformation analysis of high-altitude highway tunnels based on 3D laser scanning technology is important in the field of engineering.

To study the deformation monitoring data of a tunnel fault zone, a 3D laser scanning technique based on a point cloud was proposed in this study to obtain the relative deformation of the tunnel. Considering a highway tunnel as the research object, a 3D laser scanner was used to conduct two-phase scanning of the tunnel support and surrounding rock crossing a typical fault. The point-cloud data of the tunnel were collected during construction; denoising, registration, and surface reconstruction of the point cloud data were performed, and a 3D tunnel model was generated based on the point-cloud data. The difference between the cross section constructed based on the point cloud data and the designed cross section was analyzed by comparing the typical cross sections of the tunnel during different periods.

## 2. Theoretical Framework

### 2.1. Project Profile

The highway in Sichuan Province connects mainland China to Tibetan areas. The Kangxin Expressway runs south of Kangding City. The route starts in Kangding City and passes through Dongsheng to the Yakang Expressway. It interconnects with the Kangding G318 line with a tunnel (6670 m long) to Kangding Yulin Sima Bridge village. The route ends at Yulin New City, with a total length of 17.893 km. Its design speed is 80 km/h and its roadbed width is 25.5 m. It is covered with asphalt concrete. There are three bridges with a total length of 1.231 km, and two tunnels with a total length of 15.447 km/2; the total length of the bridges and tunnels is 16.678 km, with a bridge-to-tunnel ratio of 93.18%. Before the construction of the long tunnel, 3D laser scanning had to be performed during tunnel excavation.

### 2.2. Monitoring Principle

Three-dimensional laser scanning is a spatial data acquisition method. With high-speed laser scanning of a target object, the coordinates and reflection intensities of many points on the surface of the object can be determined [42]. Using 3D laser scanning, the 3D measurements of an entire space can be obtained using the principle of laser distance measurement, intensively recording the 3D coordinates, reflectance, and texture information of the target object’s surface. A 3D laser scanner is an active non-contact measurement system that can collect the 3D data of a large high-density space. It offers high point-measurement precision, high density of spatial point acquisition, and fast speed, and can integrate laser reflection intensity and object color information. Three-dimensional laser image data can provide research content for the identification and analysis of measurement targets. The differences between 3D laser scanning technology and traditional measurement methods are shown in Table 1.

**Figure 1:**
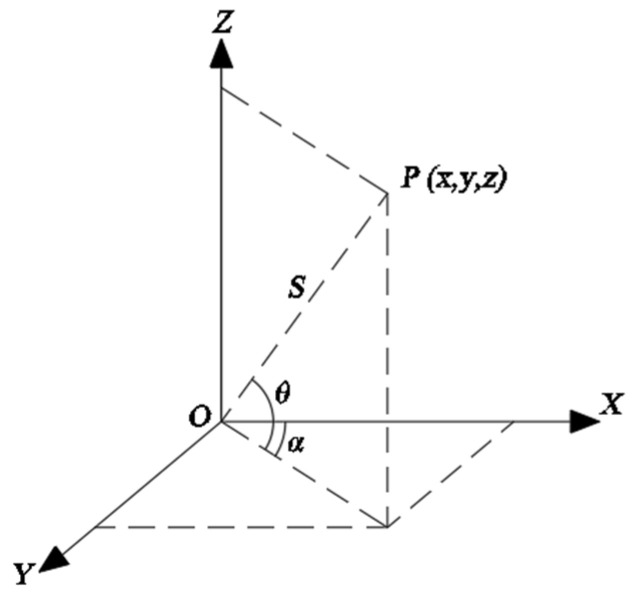
Spatial coordinate system used in the 3D laser scanner.

A 3D laser scanner obtains the distance between the sensor and the scanned object using the pulse-ranging method, and the precision clock control encoder of the scanner synchronously measures the transverse and longitudinal scanning angles of each laser pulse. The internal coordinate system of the measuring instrument is used in the 3D laser scanning measurements. As shown in Figure 1, the x-axis and the y-axis, perpendicular to the x-axis, are in the transverse scanning plane, while the z-axis is perpendicular to the transverse scanning plane. The formula for the P coordinates (X, Y, Z) of the 3D laser foot point can be expressed as follows:

(1) $$\left\{ \begin{array}{l} X=S\cos\theta \cos\alpha \\ Y=S\cos\theta \sin\alpha \\ Z=S\sin\theta \end{array}\right.$$

### 2.3. Technique Process

**Figure 2:**
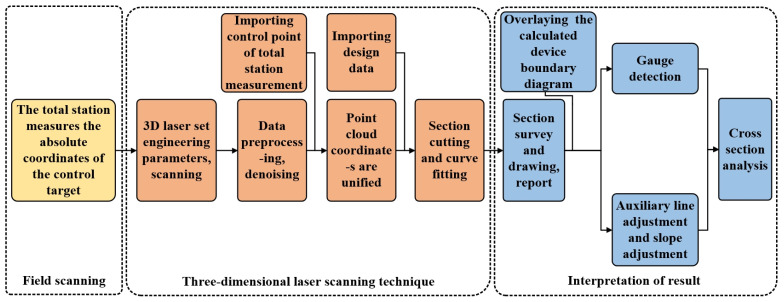
Workflow of tunnel section measurement.

In this study, a 3D laser scanning technique based on a point cloud was used to obtain the relative deformation of a tunnel. The measurement of the tunnel section encompasses three distinct stages: preparatory work, laser scanning, and subsequent result analysis. Initially, precise target coordinates are meticulously determined using a total station, ensuring utmost accuracy. Subsequently, a three-dimensional laser scanner is employed for detailed scanning and analysis. The acquired section data are then rigorously compared against the design specifications. Finally, a comprehensive analysis of the section results is conducted. The tunnel monitoring process is illustrated in Figure 2. The following section uses a highway tunnel as an example to explain the tunnel monitoring process.

## 3. Tunnel Scanning Analysis

A Leica RTC LT was used for the laser scanning of the highway tunnel used as the example, and the interior processing data of the tunnel were compared with the actual outline and data of the initial branch and secondary lining on different dates. The scanner parameters are listed in Table 2.

### 3.1. Tunnel Field Scanning

**Figure 3:**
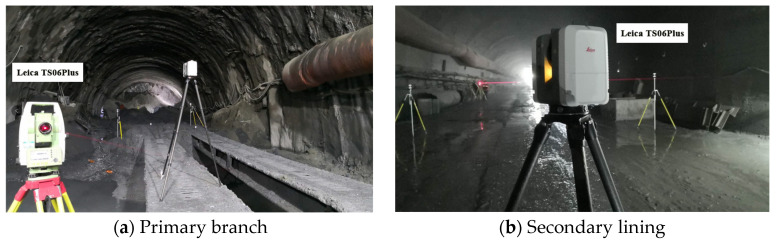

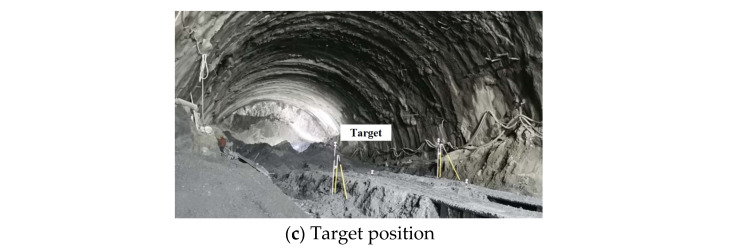
Field scanning positions.

Four stations were set up for tunnel field scanning: two stations at the initial branch position and two stations at the secondary interlining position. A target-splicing method was adopted, and the target was placed on a centering rod. The Leica TS06Plus total station was used to measure the target center coordinates in two ways, prism-free and prism-placed, to control the absolute positions of the coordinates and ensure measurement precision. The target coordinate values were measured using the total station for three days, from 29 to 31 July 2020, and the error was maintained within 2 mm. The field scanning of the positions of the primary branch and secondary lining is shown in Figure 3.

### 3.2. Tunnel Primary Branch and Secondary Lining Data Processing

**Figure 4:**
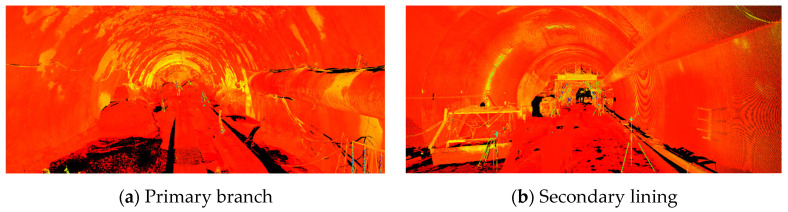
Point cloud data graph.

The tunnel point-cloud target was automatically spliced, and 1, 2, 3, 4, 5, and 6 were the control points; 3, 4, and 5 were the target splicing points, and 7 was the test point. The error at point 7 obtained through conversion was 1 mm, which complies with the field instructions. The overall point-cloud splicing and point-cloud results of the tunnel are shown in Table 3. Since the target concatenation is selected, n/a means that overlap points are not displayed. It can be observed from the error vector of all constraint points that the maximum error value does not exceed 0.002 m. Following the conversion, the stitching precision was found to be <2 mm. The cloud data of the first branch and secondary lining are shown in Figure 4.

**Figure 5:**
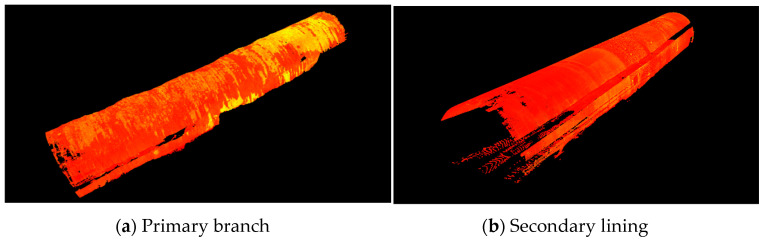
Point-cloud model diagram.

The tunnel point cloud was then denoised, the data of the first branch and secondary lining were retained, and the noise points of the ground, vehicles, people, machinery, wires, exposed steel bars, steel trestles, and fans were deleted to reduce the impact of noise data. The point-cloud model after its denoising is shown in Figure 5 (the point cloud with deletions on the right is the occluded part of the air duct).

## 4. Data Analysis

### 4.1. Analysis of the Initial Branch Section

(1) Comparative analysis of the initial branch section before and after blasting

**Figure 6:**
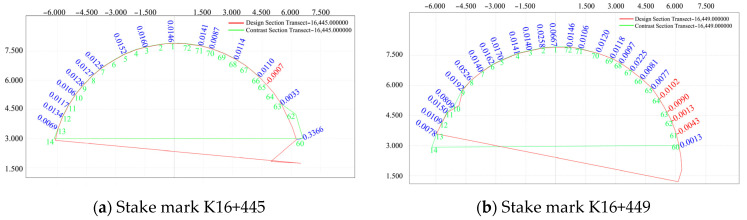

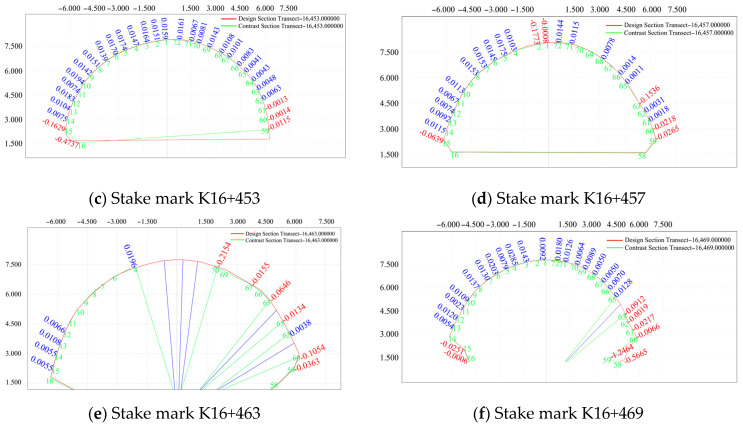
Deformation diagram of the initial surrounding rock before and after blasting.

Leica tunnel construction measurement software was used to generate section data, and a section in the pile number range of K16+435–K16+477 (2 m) was selected before and after blasting was performed on 29 July 2020. Data pertaining to the two excavation faces were collected before and after their blasting, and the section at the initial branch position of the two data periods was monitored for subsidence deformation of the convergence arch around the surrounding rock. The red values in Figure 6 represent the convergent values.

Figure 6 shows a deformation diagram of the surrounding rock of the initial branch before and after blasting for different pile numbers. The figure shows that the arch roof settlement was small and that the entire tunnel was stable both before and after the fracturing of the initial branch location. Except for a few points, the deformation on the left side of the pile number in the middle position is slightly larger than that on the right side.

**Figure 7:**
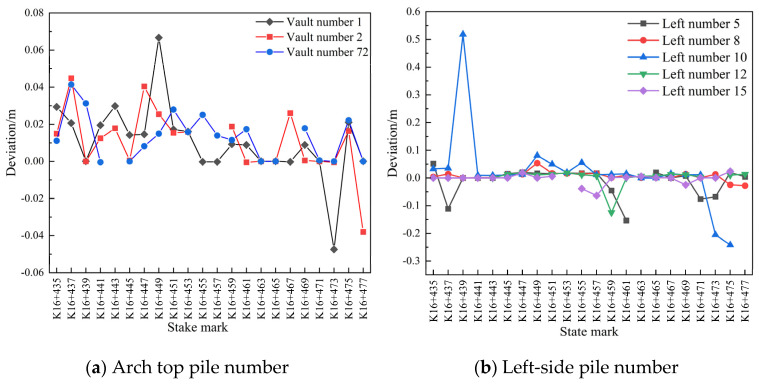

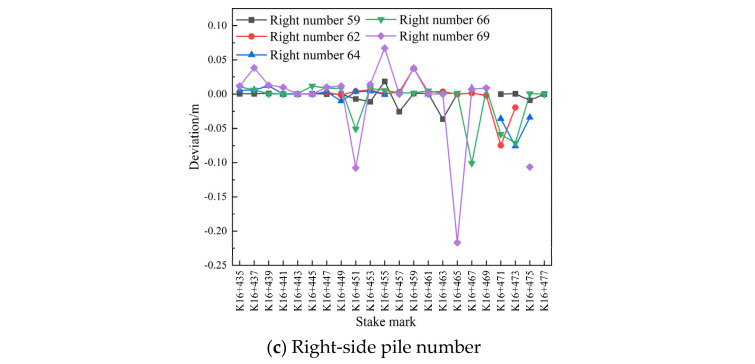
Comparison of pile number deviations.

A section in the pile number range of K16+435–K16+477 (2 m, measuring point Nos. 1, 2, 72, 5, 8, 10, 12, 15, 59, 62, 64, 66, and 69) and the section of the initial branch position before and after blasting were compared and analyzed. The related pile number deviation diagram is shown in Figure 7.

It is evident from Figure 7a that the closer each mileage of the initial branch of the left-line tunnel is to the blasting location both before and after blasting, the larger the settlement of the arch roof. The overall settlement was <0.03 m, which indicates stability, except for some mileages. As shown in Figure 7b,c, the perimeter of the initial branch of the left-line tunnel was less affected by blasting, and the individual mileages were larger compared to those of the entire tunnel, which requires attention. The overall convergence and deformation were not large and tended to stabilize.

(2) Deformation diagram of the surrounding rock of the initial section

**Figure 8:**
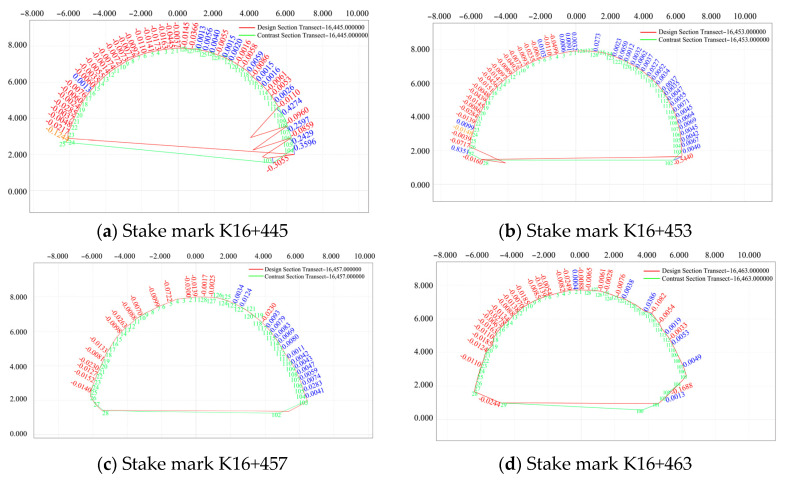

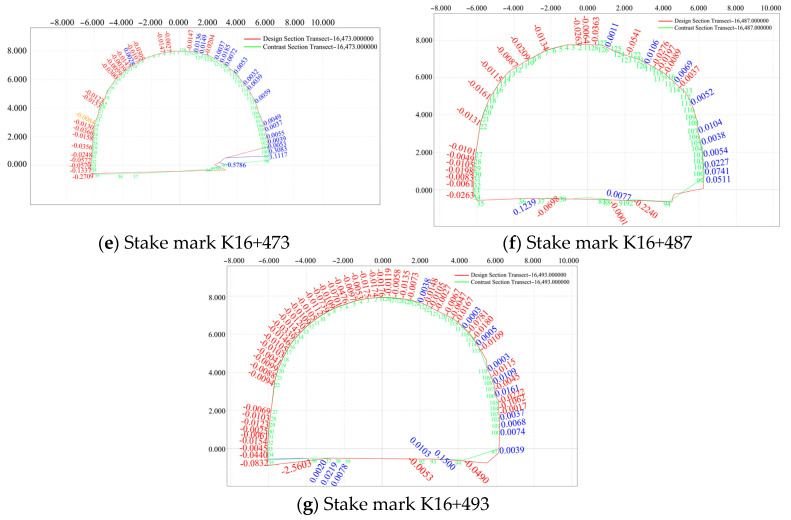
Deformation diagram of the surrounding rock obtained on 29 and 30 July 2020.

**Figure 9:**
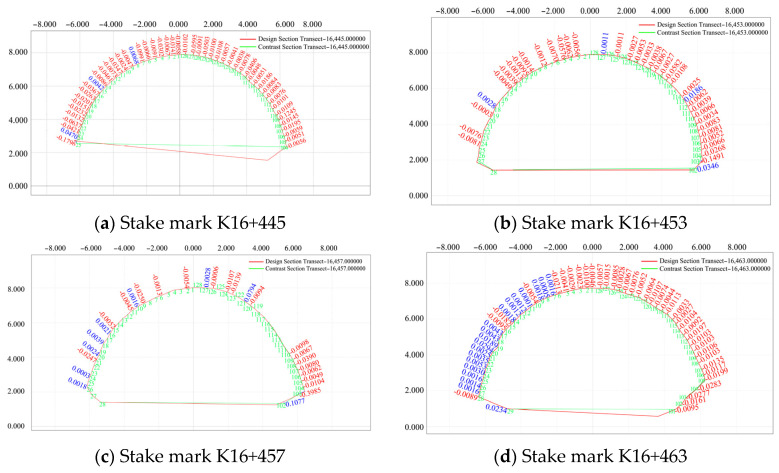

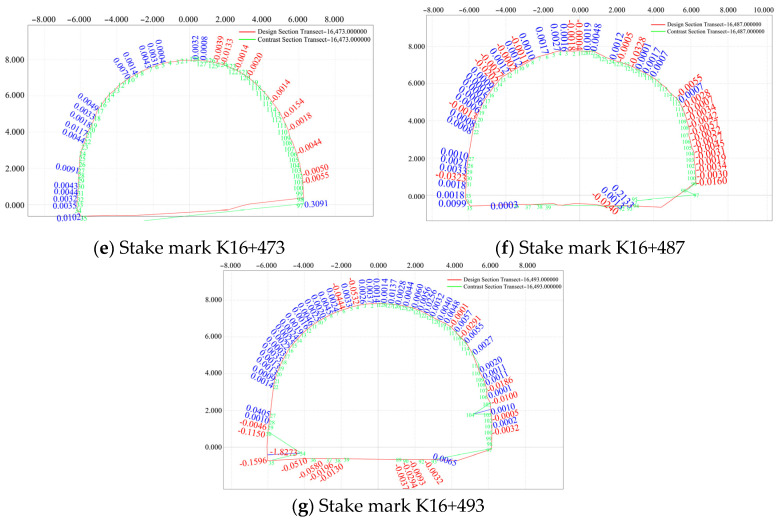
Deformation diagram of the surrounding rock obtained on 30 and 31 July 2020.

**Figure 10:**
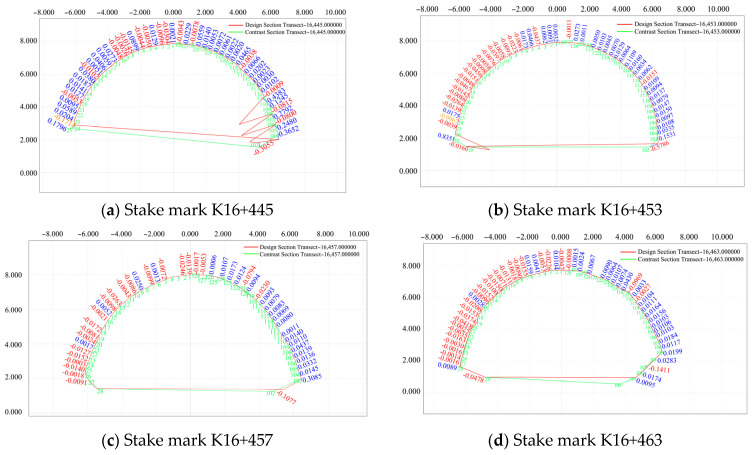

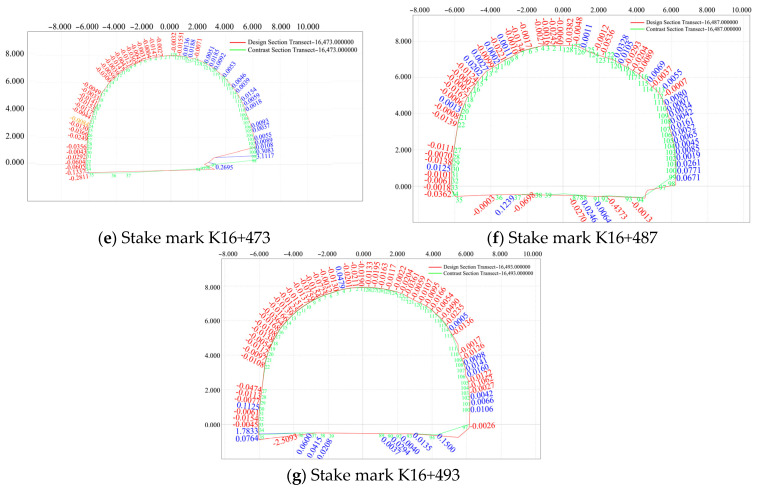
Deformation diagram of the surrounding rock obtained on 29 and 31 July 2020.

A cross section in the pile number range of K16+435–K16+493 (2 m) was observed from 29 to 30 July 2020, from 30 to 31 July 2020, and from 29 to 31 July 2020, and the data were collected at 12 and 24 h intervals, respectively. The convergence of the surrounding rock and subsidence deformation of the arch roof were monitored and analyzed for the first two days before and after blasting of the section at the position of the first branch. Deformation diagrams of the first branch surrounding the rock with the corresponding pile numbers are shown in Figure 8, Figure 9 and Figure 10.

As Figure 8 shows, the arch of the section at the initial branch location is settling down, and both sides of the perimeter are converging under mountain compression. The left side of the perimeter is deformed toward the overhead plane, whereas the right side of the perimeter is intruding into the rock mass, showing an overall trend of small deformation to the right.

As shown in Figure 9, on 30 and 31 July 2020, the arch of the section at the initial branch location had fallen downward, and the initial branches on both sides of the perimeter had invaded the rock mass. Under mountain compression, the left side of the perimeter was deformed toward the overhead plane, whereas the right side of the perimeter invaded the rock mass, showing a general tendency for slight deformation toward the right side.

As Figure 10 shows, the arch part of the section at the initial branch has a downward settlement phenomenon, and its surrounding sides gradually converge under the influence of mountain compression. In particular, deformation to the transversal surface appeared on the left side of the perimeter, while the right side of the perimeter invaded the interior of the rock mass. Overall, there is a small trend of deformation to the right.

(3) Comparison of settlement and convergence of the initial surrounding rock at different measuring points and for different pile numbers

**Figure 11:**
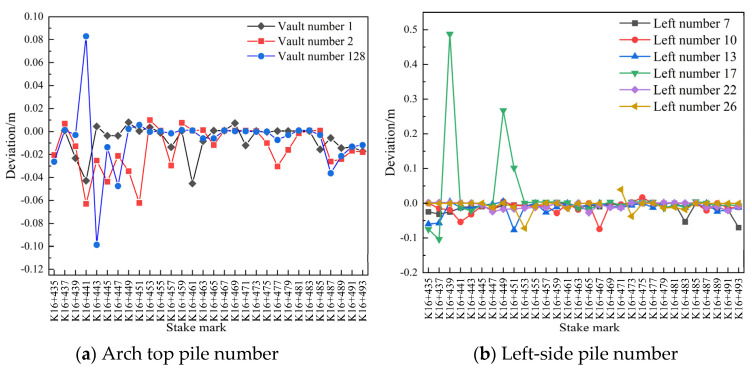

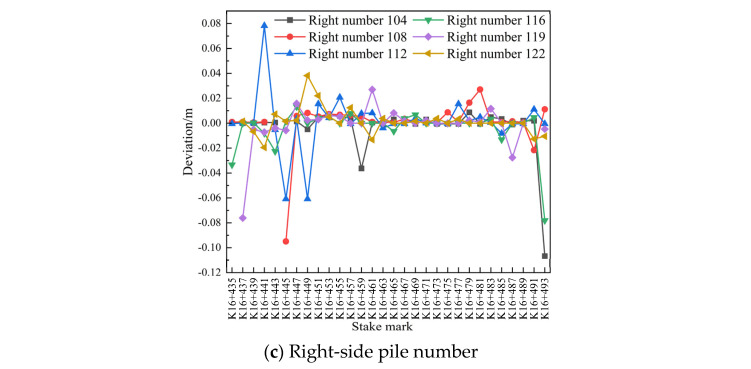
Comparison of the deviations in the pile numbers between 29 and 30 July 2020.

**Figure 12:**
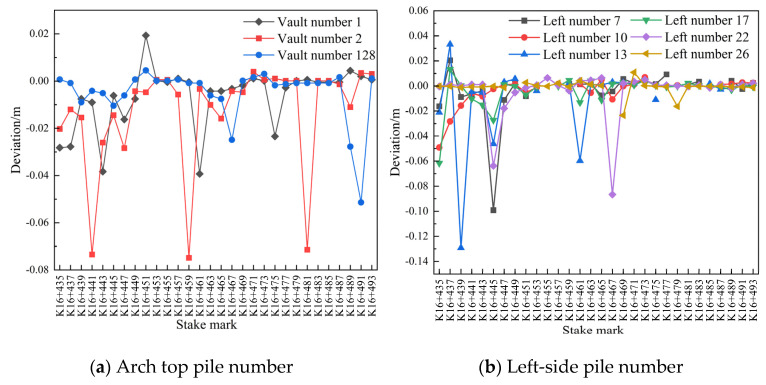

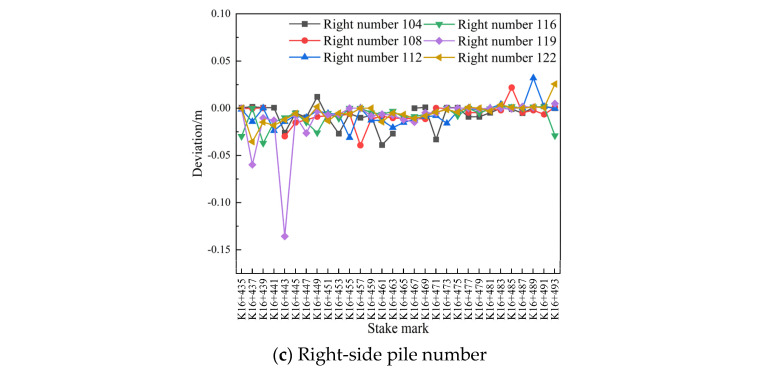
Comparison of the pile number deviations between 30 and 31 July 2020.

**Figure 13:**
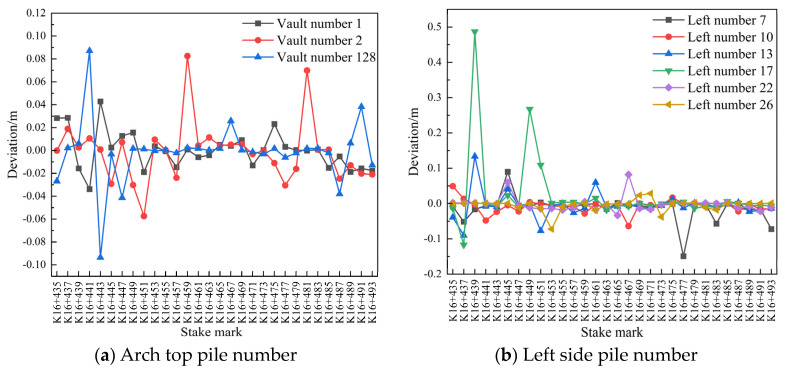

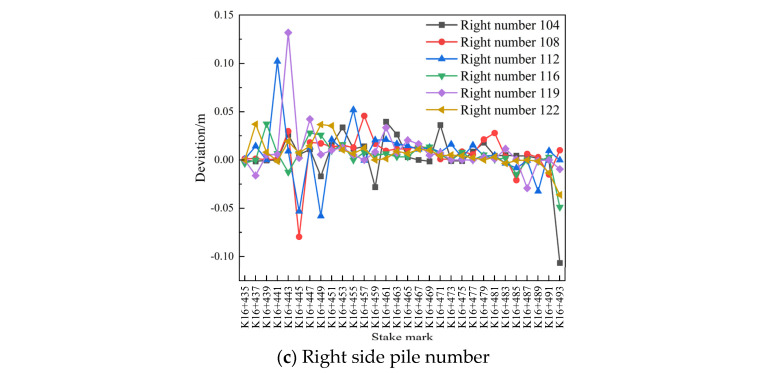
Comparison of the pile number deviations between 29 and 31 July 2020.

For the sections numbered 1, 2, 128, 7, 10, 13, 17, 22, 26, 104, 108, 112, 116, 119, and 122, the initial arch roof subsidence and peripheral displacements were compared between 29 and 30 July 2020, between 30 and 31 July 2020, and between 29 and 31 July 2020 for the pile number range of K16+435–K16+493 (2 m). Deviation diagrams of the pile numbers are shown in Figure 11, Figure 12 and Figure 13.

As Figure 11a shows, at certain measuring points within the mileage range of K16+435–K16+451, the arch settlement of the initial branch of the left-line tunnel was large, while at the rest of the measuring points within the same mileage range, the arch settlement did not exceed 0.04 m, which is noteworthy. The small arch settlement for the rest of the mileage tended to stabilize within 24 h. As shown in Figure 11b,c, the convergence around the first branch position on 29 and 30 July 2020 was generally small at most of the measuring points on both sides of the tunnel, with some measuring points showing a convergence of <0.02 m. The overall deformation remained stable.

As shown in Figure 12a, the deformation of the initial vault of the left-line tunnel K16+435–K16+447 was generally large, except at a few points where it was below 0.02 m, and the settlement of the vault at the remaining points tended to become stable within 24 h. As shown in Figure 12b,c, the overall deformation on the left side of the initial branch of the left-line tunnel was small, whereas the deformation on the right side was large. However, except at a few points, the deformation generally did not exceed 0.02 m, and the convergence on the left and right sides of the periphery tended to become stable within 24 h.

As shown in Figure 13a, the deformation of the initial vault of the left-line tunnel K16+435–K16+459 was generally large, except at a few points where it was below 0.04 m, and the settlement of the vault at the remaining points tended to become stable within 48 h. As shown in Figure 13b,c, the overall deformation on the left side of the initial branch of the left-line tunnel was small, whereas the deformation on the right side was large. However, except at a few points, the deformation generally did not exceed 0.02 m, and the convergence on the left and right sides of the periphery tended to become stable within 48 h.

(4) Chromatographic analysis

**Figure 14:**
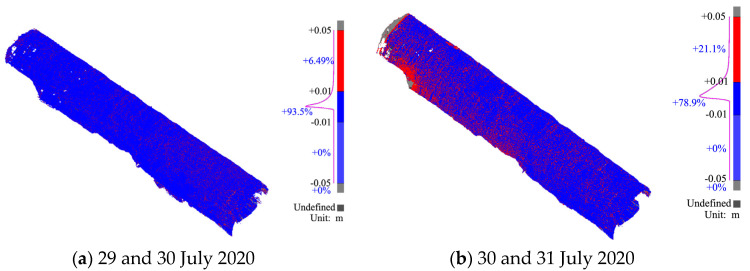
Chromatograms of the primary branch.

We used 3DR version 2020 software to obtain the chromatogram shown in Figure 14, based on the initial branch positions on 29 and 30 July 2020, and the two days before and after 31 July 2020. In the chromatogram, deviations under 0.01 m are shown in blue, and deviations exceeding 0.01 m are shown in red.

As shown in Figure 14, the arch of the section at the initial branch location settled, and both sides of the perimeter convergence were under mountain compression. The left side of the perimeter deformed toward the overhead plane, whereas the right side of the perimeter intruded into the rock mass, showing an overall trend of small deformation to the right side.

(5) Comparative analysis of the section generated by the initial branch and the designed section of the initial branch on 30 July 2020

**Figure 15:**
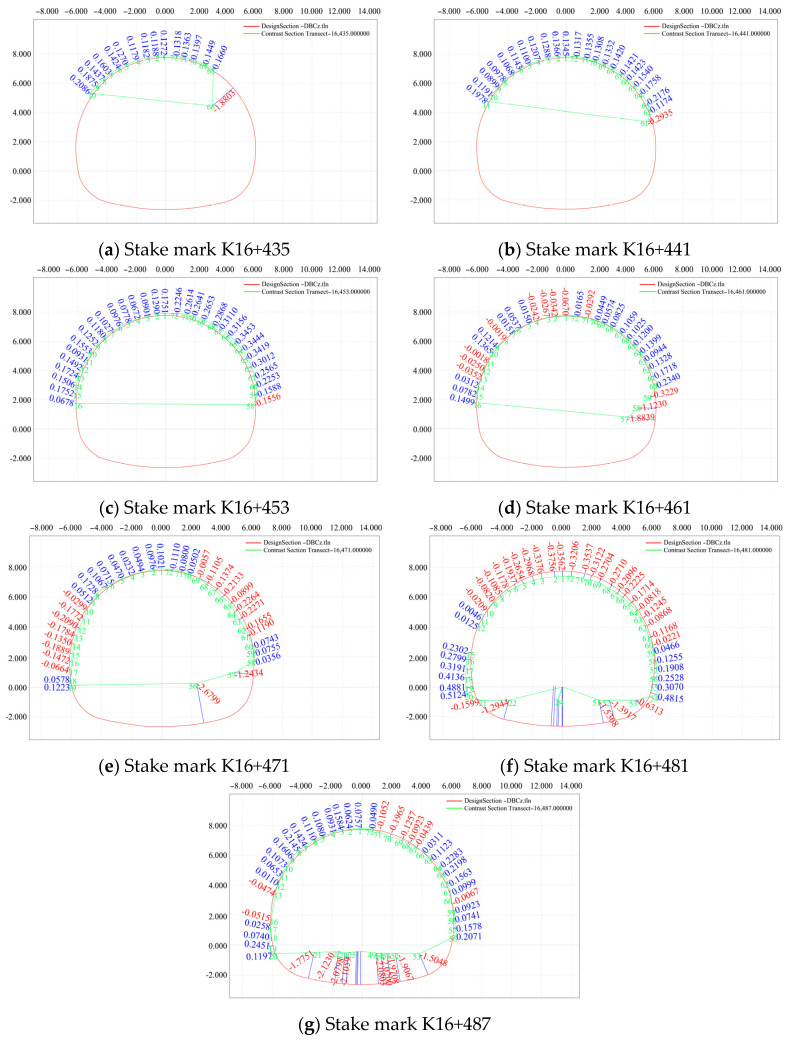
Comparison of the deformation of the first surrounding rock on 30 July 2020.

A section in the pile number range of K16+435–K16+493 (2 m) was selected on 30 July 2020, and the convergence of the surrounding rock and subsidence deformation of the arch roof were monitored and compared with those of the designed section of the initial branch. The results of this analysis are presented in Figure 15.

As is evident in Figure 15, in the presence of mountain subsidence and reserved deformation, the designed elevation of the vault was higher than its actual elevation on 30 July 2020, and the vault began to settle downward at distances father away from the initial branch position. The deformation on both sides of the periphery intruded into the rock mass, and the overall stability tended to be stable.

(6) Comparison of the settlement and convergence of the initial surrounding rock at different measuring points and for different pile numbers

**Figure 16:**
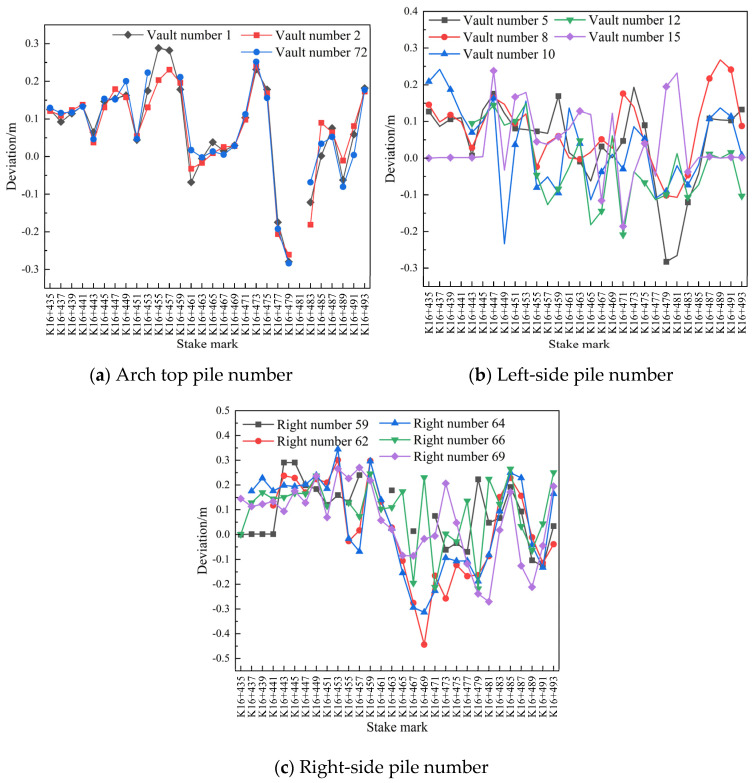
Comparison of pile number deviations on 30 July 2020.

For a cross section in the pile number range of K16+435–K16+493 (2 m, measuring point Nos. 1, 2, 72, 5, 8, 10, 12, 15, 59, 62 64, 66, and 69) , the cross section generated by the initial branch on 30 July 2020 and the designed section of the initial branch were compared and analyzed, and the results are presented in Figure 16.

As shown in Figure 16a, due to the impact of mountain settlement and reserved deformation, the actual elevation of the arch of the initial branch of the left-line tunnel was greater than the designed elevation, but no more than 0.3 m, and the overall deformation was stable. As shown in Figure 16b,c, by comparing the actual and designed data of the initial branch of the left-line tunnel, and after considering the influence of reservation deformation (≤0.3 m) , which is greater than the designed value, it can be observed that under the compression exerted by the top mountain on both sides of the surrounding areas at different mileages, the rock mass has a tendency to intrude in the tunnel.

### 4.2. Contrast Analysis of Secondary Lining Section

(1) Deformation analysis of the secondary lining

**Figure 17:**
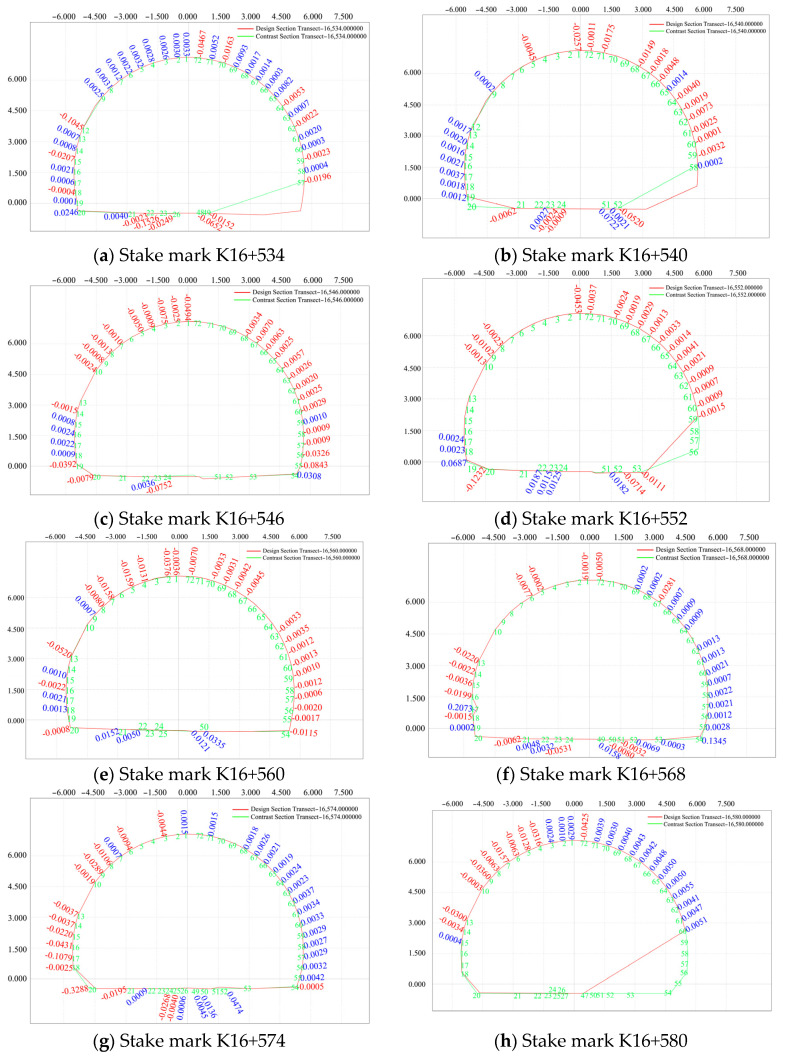
Deformation diagrams of the surrounding rock of the secondary lining.

The data of one location with representative pile numbers, collected between 29 and 31 July 2020, were selected to create deformation diagrams of the surrounding rock, as shown in Figure 17.

As shown in Figure 17, the mileage inside the tunnel presents a small deformation trend to the right side under mountain compression. Using the pile numbered K16+560 as the focal point, we observe that in the pile where the central pile number is situated to the left, the deformation of the arch is more significant than that of the left and right sides. Conversely, in the pile where the central pile number lies to the right, the deformation is more pronounced on the left and right sides compared to the arch. However, overall, the deformation remains relatively minor and exhibits a tendency towards stability.

(2) Comparison of the surrounding rock settlement and convergence at different measuring points and for different pile numbers

**Figure 18:**
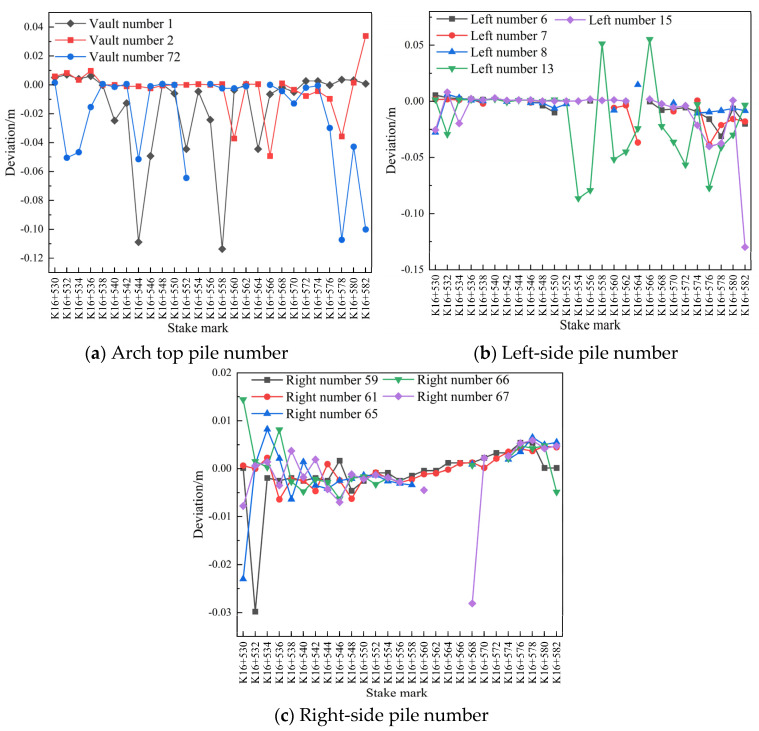
Comparison of pile number deviations between 29 and 31 July 2020.

Data comparison diagrams of measuring point Nos. 1, 2, 72, 6, 7, 8, 13, 15, 59, 61, 65, 66, and 67 in the pile number range of K16+530–K16+582, related to the subsidence and peripheral displacement of the secondary lining of the arch on 29 and 31 July 2020, are shown in Figure 18.

It is evident in Figure 18 that the settlement of the secondary lining of the arch of the tunnel was large for a few pile numbers and that the overall settlement tended to become stable within 48 h. The convergence of measuring point No. 13 on the periphery of the secondary lining of the left-line tunnel was large in the pile number range of K16+554–K16+582, and the convergence on the left and right sides of the periphery tended to become stable within 36 h.

(3) Chromatographic analysis

**Figure 19:**
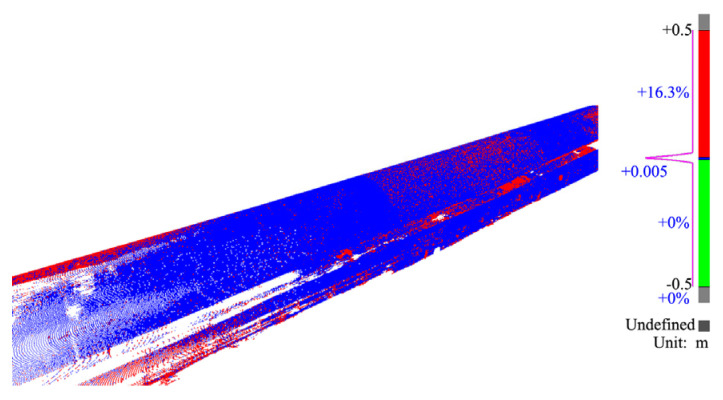
Chromatograms of the secondary lining.

We used 3DR software to obtain the chromatogram shown in Figure 19, based on the same secondary lining positions on 29 and 31 July 2020. In the chromatogram, deviations under 0.005 m are shown in blue, and deviations exceeding 0.005 m are shown in red.

(4) Comparison analysis between the design of the secondary lining and the actual section on 31 July 2020

**Figure 20:**
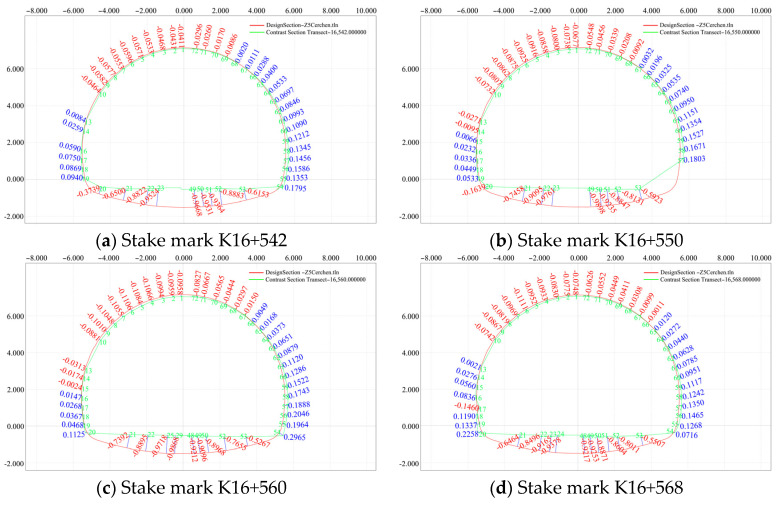

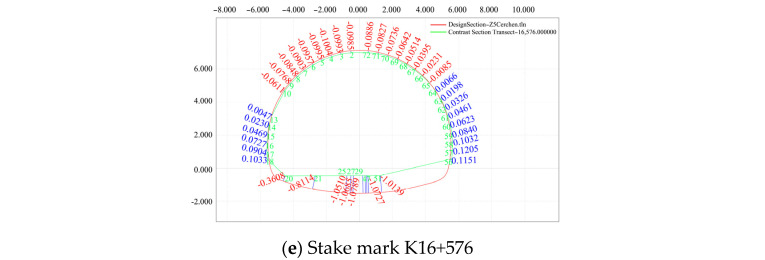
Comparative analysis of the designed section (red) and the measured section (green).

The section K16+542–K16+588 (2 m) was selected to monitor the convergence of the surrounding rock and subsidence deformation of the arch roof for typical secondary lining sections with different mileages, and a comparative analysis of the designed and actual sections of the secondary lining was performed. The analysis results are shown in Figure 20.

As shown in Figure 20, the tunnel length extends from the inside to the outside. The tunnel was mainly affected by the thrust caused by the upper slope sliding. Under the extrusion of the mountain, the arch roof descended, and the surrounding intrusion rock stratum exhibited a deformation trend on both sides.

**Figure 21:**
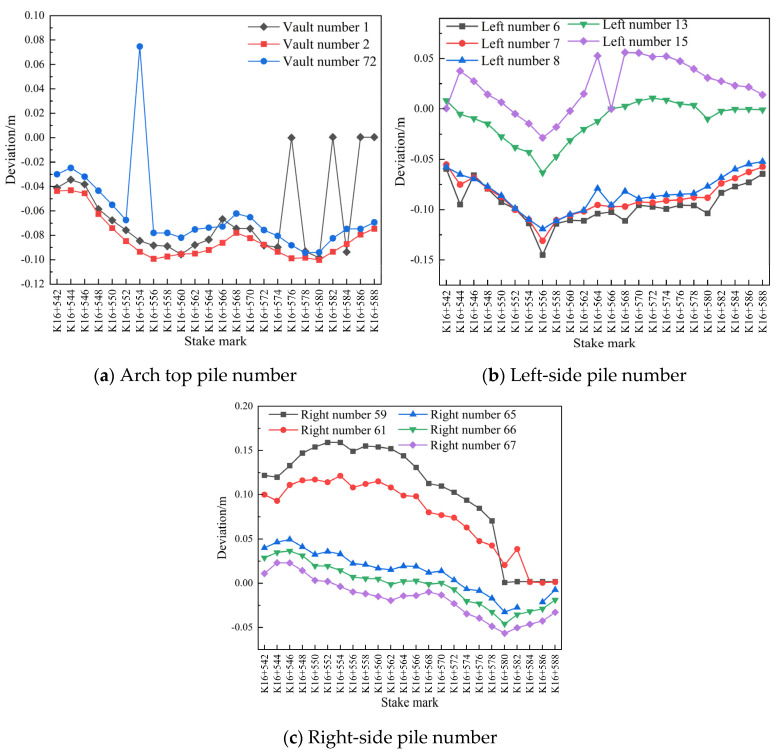
Comparison of the pile number deviations on 31 July 2020.

For section Nos. 1, 2, 72, 6, 7, 8, 13, 15, 59, 61, 65, 66, and 67 in the pile number range of K16+542–K16+588 (2 m), the design of the secondary lining of the arch roof subsidence and peripheral displacement were compared with the actual section data on 31 July 2020. The pile number deviations are shown in Figure 21.

As shown in Figure 21, compared with the actual and designed values of the secondary lining of the arch of the left-line tunnel, each mileage at different measurement points exhibited the same downward settlement trend, and at mileages of K16+560 and K16+580, the settlements were large; however, the overall deformation of the surrounding rock tended to be stable and remained within 0.12 m.

## 5. Discussion

A workflow for 3D laser scanning in tunnel monitoring is proposed in this study and was analyzed in combination with actual engineering practices. Based on the construction schedule of a highway tunnel crossing a fault, a 3D laser scanner was used to conduct initial and secondary scanning of the tunnel support and surrounding rock crossing a typical fault; collect the tunnel point-cloud data during construction, denoising, registration, and surface reconstruction; and generate a 3D tunnel model based on the point-cloud data. The difference between the cross section constructed based on the point-cloud data and the designed cross section was analyzed by comparing the typical cross sections of different periods. The deformation of the surrounding rock at the position of the first branch and the secondary lining shows a trend of smaller deformation to the right. These study findings indicate that during the tunnel’s construction, the influence of the surrounding rock is mainly observed on the left side, while the right side remains stable. This deformation trend may be related to the tunnel construction method, geological conditions, and blasting operations. At different pile numbers and measuring points, the settlement of some arch mileage is relatively significant. This large settlement may be owing to uneven geological conditions, improper operation during construction, or other external factors. However, it is worth noting that these settlement values tend to be stable within a certain time range, indicating that the tunnel structure was gradually adapting and stabilizing. Except for some specific points, the deformation of the left and right sides is maintained within 0.02 m. Thus, the overall stability of the tunnel structure was good, and the deformation was within a small range. However, for individual large deformation points, further inspection and analysis would be required to determine their causes and implement appropriate treatment measures. The actual elevation of the first branch arch of the tunnel (<0.3 m) was greater than the designed elevation. This difference could be due to errors in the construction process, the impact of measurement precision, or even other factors. However, this difference was within the acceptable range and did not significantly affect the safety or stability of the tunnel. The actual and designed settlement values of the secondary lining of the arch of the tunnel showed the same downward trend at different measuring points of each mileage. This trend may be related to the deformation of the primary branch, construction quality of the secondary lining, or changes in the geological conditions. To ensure the safety and stability of the tunnel, continuous monitoring and analysis of the secondary lining settlement and the implementation of corresponding treatment measures would be necessary. In summary, the monitoring results of the tunnel section and the comparison of the actual and designed sections of the primary and secondary linings show that the tunnel structure was affected during the construction process to a certain extent, but the overall stability and deformation control were within their acceptable ranges. To ensure the safety and stability of the tunnel, strengthening of the monitoring and analysis and the implementation of the corresponding treatment measures would be necessary. The structural analysis showed that the proposed 3D laser scanning technology has not only the same measurement precision as the traditional measurement technology but also several advantages, such as high data acquisition efficiency and strong data comprehensiveness. The visualization of tunnel deformation during monitoring was realized using 3D laser scanning technology, and the obtained tunnel deformation results are close to the actual deformation results. Deformation analysis based on the section curve, tunnel vault, and measurement points on both sides can reflect the deformation of the tunnel structure at both local and overall levels. Using 3D laser point-cloud technology, a 3D chromatogram of the entire deformation of the tunnel and a 2D cross section of any position in the tunnel can be obtained. This provides a scientific and feasible scheme for monitoring tunnel structure deformations.

Wan et al. [43] extracted feature points based on section location to analyze the convergence of a tunnel diameter and the trend of the total displacement of the section location to overcome the challenges faced in performing accurate deformation analysis. These challenges result from failure to determine the datum surface of the tunnel model through single-point scanning. The test results showed that the analysis results could be improved by using the section characteristic points to analyze the tunnel deformation. Liu et al. [44] analyzed and summarized the principles and applications of 3D laser scanning technology. The application of 3D laser scanning technology to determine tunnel section deformation revealed that 3D laser scanning technology has the advantages of high precision, high efficiency, and stable operation. This study focused on the application of 3D laser scanning technology to the full-section deformation of high-altitude fault tunnels. The utilization of 3D laser scanning technology is not merely straightforward but also endowed with waterproof and moisture-resistant qualities. These features enable it to operate with remarkable stability across a diverse array of environments, including those that are wet or dust-laden. Furthermore, the robust environmental adaptability of this equipment allows it to excel in complex settings such as field measurements. Nevertheless, despite its inherent waterproof and dustproof capabilities, it is worth noting that in extreme or exceptional environmental conditions, supplementary protective measures may still be necessary to safeguard the smooth operation of the equipment and maintain the precision of scanning outcomes. Therefore, when deploying 3D laser scanning technology, it is advisable for users to judiciously select and employ the appropriate equipment based on specific environmental conditions and usage requirements, thus ensuring optimal scanning performance. Owing to the differences in tunnel lengths and deformation rates, the monitoring frequency and coverage could be limited. A monitoring frequency that is too low will not be able to capture the deformation of the tunnel in time, while a monitoring frequency that is too high will increase the cost and workload. Three-dimensional laser scanning technology also has many error sources, including instrument performance, the scanning environment, the scanning point-cloud density, and the scanning target material. In follow-up work, the influence of these errors on the results can be reduced by improving the algorithm and optimizing the data processing process, so as to improve the precision and reliability of the data.

## 6. Conclusions

In this study, taking the Kangxin Expressway tunnel as an example, the deformation characteristics of a tunnel were studied using 3D laser scanning technology. The conclusions of the study are as follows.

(1) The monitoring results of the tunnel section indicate that the distance between the first branch and secondary lining was from the inside to the outside and that the deformation of the surrounding rock exhibited a tendency for small deformations toward the right side. At different measuring points and with different pile numbers, the settlement at individual mileages of the vault was large but tended to become stable within a certain time range. Except for a few points, the deformation of the left and right sides did not exceed 0.02 m.
(2) A comparison of the actual and designed sections of the first branch and the secondary lining showed that the surrounding rock arch had a downward settlement trend and that the deformation on both sides of the surrounding rock intruded into the rock mass. The actual elevation of the first branch arch (<0.3 m) was greater than the designed elevation. The actual monitoring and designed values of the secondary lining of the arch of the tunnel also showed a tendency for downward settlement at the different measuring points of each mileage.
(3) Based on the chromatographic analysis conducted on the initial branch section, it is evident that the deformation deviation of the majority of points falls within a narrow range of 0.01 m, indicating a relatively stable condition. Only a handful of points exhibit slightly larger deformation deviations. Conversely, in the secondary lining section, the deformation deviation of most points is even more constrained, staying within the range of 0.005 m, signifying an overall stable structural state. Nevertheless, it is noteworthy that certain points still surpass the threshold value of 0.005 m. Therefore, during subsequent monitoring and maintenance activities, special attention should be paid to these threshold-exceeding points to ensure the integrity and safety of the structure.

## Figures and Tables

**Table 1:** The differences between 3D laser scanning technology and traditional measurement methods.

Comparative Item	Traditional Measurement Method	Three-Dimensional Laser Scanning Technique
Tool	Tape measure; laser rangefinder; total station; drawing	Three-dimensional laser scanner
Measurement mode	Contacting, close measurement; light affected	Completely non-contact; Remote measurement; not affected by light; works day and night
Field drawing manuscript	Need	No need; automatically generates 3D data
Measurement efficiency	Low efficiency; only the distance from point to point can be measured; high labor intensity	Fast sampling rate; single-station panoramic scan in 1 min
Degree of safety	High risk factor; great limitation	Non-contact measurement; ensures personnel safety

**Table 2:** Technical parameters of the Leica RTC LT scanner.

Scanned Area	Scanning Speed	Resolution Ratio	Precision		
			Angular Precision	Ranging Precision	Range Noise
0.5 m–50 m	1,000,000 points/s	3.1 mm @ 10 m	18″	1 mm + 10 ppm	0.4 mm @ 10 m

**Table 3:** Stitching precision after conversion.

Constraint ID	ScanWorld	ScanWorld	Type	Status	Weight	Overlap Points	Error (m)	Error Vector (m)
1	Job 007-Setup 001	Job 007-Setup 002	Coincident: Vertex-Vertex	On	1.0000	n/a	0.002	(−0.001, 0.000, 0.001)
7	Job 007-Setup 001	Job 007-Setup 002	Coincident: Vertex-Vertex	On	1.0000	n/a	0.001	(−0.001, 0.000, −0.001)
1	Job 007-Setup 001	20200729kzd.txt (Leveled)	Coincident: Vertex-Vertex	On	1.0000	n/a	0.002	(−0.002, −0.001, 0.001)
3	Job 007-Setup 001	20200729kzd.txt (Leveled)	Coincident: Vertex-Vertex	On	1.0000	n/a	0.002	(0.002, −0.001, 0.001)
4	Job 007-Setup 001	20200729kzd.txt (Leveled)	Coincident: Vertex-Vertex	On	1.0000	n/a	0.002	(0.002, 0.000, 0.001)
7	Job 007-Setup 001	20200729kzd.txt (Leveled)	Coincident: Vertex-Vertex	On	1.0000	n/a	0.001	(0.001, 0.001, 0.001)
6	Job 007-Setup 002	20200729kzd.txt (Leveled)	Coincident: Vertex-Vertex	On	1.0000	n/a	0.002	(−0.002, −0.001, −0.001)
7	Job 007-Setup 002	20200729kzd.txt (Leveled)	Coincident: Vertex-Vertex	On	1.0000	n/a	0.002	(0.001, 0.001, 0.001)
1	Job 007-Setup 002	20200729kzd.txt (Leveled)	Coincident: Vertex-Vertex	On	1.0000	n/a	0.002	(−0.001, −0.001, −0.001)
5	Job 007-Setup 002	20200729kzd.txt (Leveled)	Coincident: Vertex-Vertex	On	1.0000	n/a	0.003	(−0.001, 0.002, 0.001)

## Data Availability

The data presented in this study are available on request from the corresponding author.
